# Micro and macro structural brain plastic changes induced by sexual experience in male rats

**DOI:** 10.1371/journal.pone.0334959

**Published:** 2025-10-28

**Authors:** Zacnite Mier-Quesada, Lorena Gaytán-Tocaven, Alberto Prado, Raúl G. Paredes

**Affiliations:** 1 Instituto de Neurobiología, Universidad Nacional Autónoma de México, Querétaro, México; 2 Escuela Nacional de Estudios Superiores, Unidad Juriquilla, Universidad Nacional Autónoma de México, Querétaro, México; Nathan S Kline Institute, UNITED STATES OF AMERICA

## Abstract

Sexual behavior induces brain plastic changes such as neurogenesis, but few studies have evaluated possible changes in synaptic plasticity produced by sexual experience. In the present study, we assessed whether two aspects of sexual behavior in male rats, sexual incentive motivation and sexual execution in a partner preference test, could induce micro and macrostructural changes in brain regions involved in controlling sexual behavior belonging to the socio-sexual behavior network and the mesolimbic reward circuit. The microstructural changes were evaluated by synaptophysin immunofluorescence expression. We assessed the macrostructural changes using manganese-enhanced magnetic resonance imaging and volume changes by magnetic resonance imaging. Our results indicate that the mesolimbic reward circuit underwent plastic changes at the level of synaptophysin expression, mainly in the partner preference test group. In the socio-sexual behavior network circuit, an increase in brain activation was observed primarily in the sexual incentive motivation group. When analyzing the activation of the whole brain, the statistical map showed a significant increase in weeks 5 and 10 compared to week 1 in the sexual incentive motivation group. The results confirm that different neuroplastic changes, including synaptophysin expression, brain activation, and volume changes, occur during the acquisition of sexual experience.

## Introduction

Motivated behaviors satisfy biological needs, promoting the survival of individuals and their offspring. These behaviors include sleeping, mating, eating, drinking, and parenting [1]. The motivation to display these behaviors depends on the arousal level, which is related to how vigorously the subject pursues a goal [1]. The motivated behaviors trigger physiological responses, including a series of internal adjustments [2]. Usually, female rats spend more time near sexually active males, while males spend more time near estrous females [2]. Sexual behavior is a motivated behavior because it is voluntary and unpredictable. Different studies have demonstrated that, in rodents, the selection of a suitable sexual partner depends on the processing of chemosensory-relevant olfactory signals [3,4] by a large olfactory system crucial for reproduction [5].

Several brain regions are involved in the control of sexual behavior, including those that belong to the Socio-sexual Behavior Network (SBN) and the Mesolimbic Reward System (MRS), which regulate behavior and evaluate the importance of stimuli and behavioral output [6]. The SBN is associated with different forms of behavior, such as sexual behavior, aggression, and parental care [6]. It is composed of the olfactory bulb (OB), the ventromedial hypothalamus (VMH), the medial preoptic area (MPOA), the bed nucleus of the stria terminalis (BNST), and the amygdala (AMG). On the other hand, the brain regions that comprise the MRS include the nucleus accumbens (NAcc), the hippocampus (HPO), the striatum (Str), and the ventral tegmental area (VTA). The MRS is a remarkably conserved neural system that plays a crucial role in mediating behaviors related to the motivational aspects of social interaction, reward processing, and positive reinforcement. The SBN and the MRS share structures such as the BNST and the AMG [6,7].

Sexual behavior is not a static process; it changes as the animal gains sexual experience [8]. For example, as males acquire sexual expertise, they are more effective in expressing mating behavior. This effectiveness could be associated with changes in synaptic plasticity, such as the formation of new synapses (synaptogenesis), new neurons (neurogenesis), and changes in the activation of different brain regions. Sexual experience can induce physiological and morphological changes associated with synaptic formation and remodeling (plasticity) [9]. We have shown that sexual behavior increases synaptophysin (Syp) expression in the OB [10] in mice and induces neurogenesis in the OB system in male and female rats [11].

MEMRI contrast is proportional to the accumulation of Mn2+ in different tissues. MnCl2 can be injected intravenously, intraperitoneally, or subcutaneously. The successful application of this technique depends on the appropriate delivery of ionic doses to the regions of interest. In our lab, we found that a subcutaneous dose of 16 mg/kg (MnCl2) induces the best contrast, minimizing toxicity without affecting the display of motivated behaviors, including sexual behavior [12]. Under these circumstances, contrast enhancement reaches its equilibrium 24 h following MnCl2 administration [12].

Recently, in our laboratory, using the Manganese-Enhanced Magnetic Resonance Imaging (MEMRI) technique, we observed an increase in signal intensity or activation in brain regions and circuits involved in the control of sexual behavior after mating in female and male rats [13,14]. In the present experiment, we evaluated whether sexual motivation and/or the execution of sexual behavior could induce plastic changes such as synaptogenesis, determined by Syp expression, a protein used as a marker of synaptic plasticity. We also tested whether sexual behavior increases neuronal activation through the incorporation of manganese, reflected in an increase in voxel intensity. Additionally, we analyzed volume changes in different brain regions that control sexual behavior using magnetic resonance imaging (MRI) techniques. For this purpose, we tested male rats in the sexual incentive motivation test (SIM) and the partner preference test (PPT) to determine Syp expression using immunofluorescence and stereology techniques. We also used two MRI techniques, MEMRI and FSL’s Bias tool, to evaluate increases in signal intensity (activation) and volume changes resulting from the acquisition of experience in the SIM and PPT. Each of the experiments carried out in the present study was intended to analyze neuroplastic changes at 3 different levels to evaluate sexual experience-induced changes after the subjects gained experience in the PPT group and the SIM group.

## Materials and methods

### Subjects

Male (n = 30) Wistar rats (300–350 gr), sexually naïve were used. The animals were maintained at a constant room temperature of 22–24 °C on a reverse 12h/12h light/dark cycle with water and food available *ad libitum*. They were randomly assigned to one of three groups: 1) the control group (CTRL), 2) subjects tested in the SIM tests, and 3) subjects tested in the PPT. Lamorte’s power analysis was performed to estimate the sample size, with a p < 0.05 and 95% power. As stimulus animals, we used ovariectomized female rats and sexually experienced male rats. Stimulus females were brought into estrus by injections of estradiol benzoate (25 μg/kg) and progesterone (1 mg/kg) 48 and 4 hours before mating tests, respectively.

All experiments followed the “Reglamento de la Ley General de Salud en Materia de la Investigación para la Salud” of the Mexican Health Ministry and NIH guidelines. The Animal Care Committee of the Instituto de Neurobiología approved the protocol (097A). Every effort was made to minimize the number of animals used and their suffering. Surgery was performed under sodium pentobarbital anesthesia, and during MRI image acquisition, the animals were anesthetized with 2–2.5% isoflurane. We monitored the vital signs throughout the scan and observed the animals until they were fully awake.

### Behavioral tests

#### Sexual Incentive Motivation (SIM).

**Fig 1:**
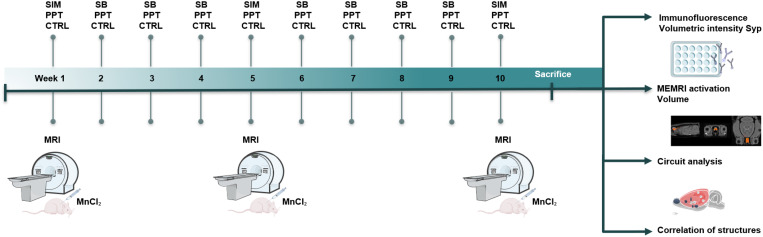
Timeline of the sequence of tests. Representative timeline diagram to assess Sexual Incentive Motivation (SIM) and Partner Preference Test (PPT) during the 10 weeks of testing. Subjects were tested for SIM in weeks 1, 5, and 10. In the other weeks, males mated (SB) to gain sexual experience. The PPT was conducted once a week for 10 weeks. The scanning to obtain MEMRI data was done in weeks 1, 5, and 10. Subsequently, we performed an immunofluorescence protocol and analysis of activation, volume, circuitry, and structural correlation.

The SIM was evaluated in a rectangular, central, black acrylic cage (100 x 50 cm) with two additional compartments located in the diagonally opposite corners (20 x 30 cm). Before starting each test, the compartments were cleaned with 70% alcohol. A stimulus animal was placed in each lateral compartment: a sexually receptive female and, in the opposite compartment, a sexually experienced male. In the SIM test, the subject can smell, hear, and see the stimulus subjects but has no physical contact since the compartments are separated by a wire mesh [15,16]. An incentive zone was established within the central compartment (20 x 30 cm) next to each stimulus compartment. During 10 min, we registered the time the experimental subject spent in each incentive zone. Subjects were tested for SIM in weeks 1, 5, and 10. During the other weeks, subjects were mated once a week with a sexually receptive female in rectangular mating cages in 30 min sessions to gain sexual experience (see mating tests). A timeline of the sequence of tests is depicted in Fig 1.

The SIM test was carried out interspersed with mating sessions because the animals need to acquire sexual experience, just like those in the PPT group, and to minimize the possibility that consecutive SIM sessions could reduce the incentive value of the stimulus female.

#### Partner Preference Test (PPT).

The PPT was performed in a rectangular wooden cage (32 x 36 x 34 cm) divided into three equal compartments by wooden walls. These compartments are connected by removable doors (10 x 10 cm), allowing the experimental animal to move from one compartment to the other. Before placing each animal, the compartments were cleaned. A stimulus animal was placed in each lateral compartment: a sexually receptive female and a sexually experienced male. Stimulus animals were tied with a harness, which allowed them to move only within their compartment and display sexual behavior. The experimental subjects were initially placed in the central compartment; the doors were then opened, allowing the experimental animals to move freely between compartments. During 15 min we registered the time spent in each compartment and the sexual behavior displayed with each stimulus animal. We registered the number and latencies of mounts, intromissions, and ejaculations. Subjects were tested once weekly for 10 weeks.

#### Mating tests.

Mating tests were conducted in rectangular, clear acrylic boxes measuring 60 x 40 x 30 cm. At the beginning of the test, the experimental male was placed inside, and 5 minutes after habituation, a sexually receptive stimulus female was introduced. The test lasted 30 minutes, and the number and latencies of mounts, intromissions, and ejaculations were recorded.

#### CTRL group.

The CTRL group did not undergo the behavioral tests. During the 10 weeks of the study, they were placed in the mating cages (see mating test) without stimulus animals. Before placing each animal, the mating cage was cleaned with 70% alcohol.

### Histology

**Fig 2:**
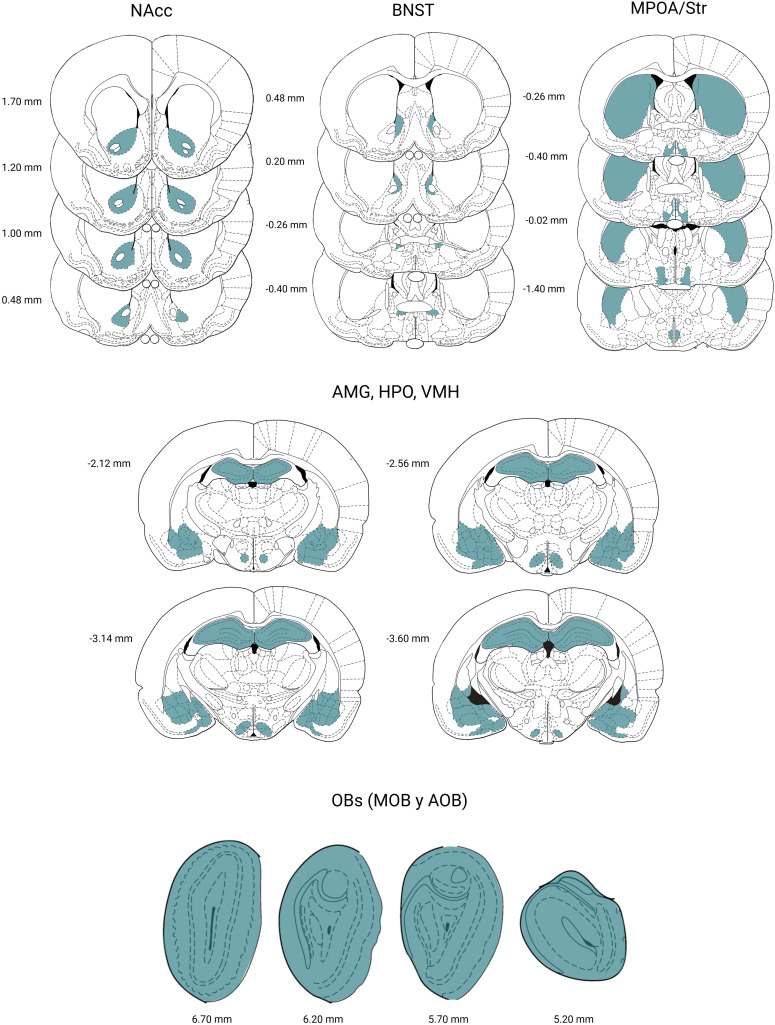
Rat brain sections from the evaluated regions. Coronal sections of the rat brain showing in blue the ROIs evaluated in this study: nucleus accumbens (NAcc), bed nucleus of the stria terminalis (BNST), medial preoptic area (MPOA), striatum (Str), amygdala (AMG), hippocampus (HPO) divided into Ammon’s horn 1 (CA1), Ammon’s horn 3 (CA3), dentate gyrus (DG), ventromedial hypothalamus (VMH), olfactory bulbs (OBs) divided into main olfactory bulb (MOB) and accessory olfactory bulb (AOB). Modified from Paxinos and Watson (1997). Created by BioRender.com.

After the last behavioral test on week 10, we deeply anesthetized the males with an intraperitoneal overdose of sodium pentobarbital and perfused them intracardially with phosphate buffered saline (PBS) and paraformaldehyde. We removed the brains and left them in a 30% sucrose solution. Using a cryostat, we cut the brains into 30 µm sections, including the OBs, BNST, MPOA, VMH, AMG, NAcc, Str, and the HPO. We selected 4 slices of different coordinates per rat according to the regions of interest (ROIs) to obtain a volumetric representation of each ROI (Fig 2).

### Immunofluorescence

All sections were incubated overnight at 4°C with the primary polyclonal antibody anti-rabbit (anti-Syp, 1:1000, PA5–27286, Invitrogen, USA). Sections were later washed in PBS (0.1 M, pH 7.2) and incubated for 90 minutes in a secondary antibody (Alexa Fluor 568 goat anti-rabbit, 1:1000, Invitrogen, USA). After that, sections were rewashed and incubated in Hoechst (1:1000, 33258, Sigma-Aldrich) for 10 minutes. At the end of the procedure, brain slices were mounted and covered using AquaPoly/Mount (Polyscience, Inc.) [10]. Subjects from the different groups were processed in parallel.

### Confocal microscopy

Images were obtained in a Zeiss LSM 700 confocal microscope with the 10X objective and the ZEN 2012 software. The laser settings were kept at 555 nm 2% (red) for Syp and 490 nm 2% (blue) for Hoechst. The capture parameters were established in the CTRL group to avoid bias and applied to the experimental groups. The ROIs were delimited in each image (Fig 2), and intensity data were obtained. We quantified the signal intensity bilaterally for each section and calculated the mean. To obtain the final volumetric Syp intensity, the data were normalized by subtracting the Syp intensity of each ROI from the intensity of an area where no changes in Syp intensity would be observed; in this case, the anterior commissure or corpus callosum was used. The z-stack of the ZEN program of the confocal microscope was used to select a 3 µm slice in the z axis for each coordinate. Four photomicrographs of each slice were obtained. Then, we average the intensity values of the 4 sections of the 4 coordinates selected per rat for each ROI and determine the volumetric value. We obtained a total of 1080 images from all the ROIs. Although there was no inter-rater reliability for synaptophysin quantification, the control group was always analyzed in relation to the experimental groups, which provides an explicit reference and thus reduces the influence of potential bias in data interpretation, thereby minimizing potential limitations.

### Manganese chloride administration

For the MEMRI technique, manganese chloride (MnCl2) (SIGMA-ALDRICH, Product number 244589, St. Louis) was dissolved in saline (MnCl2, 16 mg/kg/10 ml) and injected subcutaneously (s.c.) at a dose of 16 mg/kg. Previous studies have shown that this dose has no toxic effects and produces images of excellent quality for analysis [13,14]. In all experiments, MnCl2 was injected 24 h before the behavioral evaluation in weeks 1, 5, and 10. Immediately after the behavioral tests, we took the experimental males to an adjacent room for scanning. As described above, during MRI image acquisition, the animals were anesthetized with 2–2.5% isoflurane, and their vital signs were monitored. The acquisition of images was performed in weeks 1, 5, and 10, allowing us to evaluate changes in brain activation associated with sexual behavior over time. We used the Software Library v6.0 (FSL), the Advanced Normalization Tools (ANTs, v2.1), and the DenoiseImage tool for denoising. Using the multivariate template construction tool (ANTs) [17], we created a template of the T1-weighted image brain average from the set of scans from week 1. After creating the template, spatial normalization was performed on the denoised images using the ANTs Registration SyN tool. The scans from weeks 1, 5, and 10 were then registered to the template. We then used the tool fslmerge from the FMRIB Software Library v6.0 to merge all the aligned scans. Images with artifacts or bad co-registration were eliminated. The regions of interest were selected based on the Paxinos and Watson atlas and standardized with respect to the Harderian gland, a structure unrelated to sexual behavior. The experimenters were not blinded to group assignment during image acquisition and analysis. However, the experimental groups were always analyzed with respect to their respective controls. Moreover, at the national magnetic resonance imaging laboratory LANIREM, where the images were obtained, the technicians acquiring the images were unaware of which group each animal belonged to, thereby reducing the influence of potential bias in data analysis and interpretation.

### Structure volume

We analyze the volume by structure, comparing the groups through the FSL program using the N4BiasFieldCorrection tool. Spatial normalization was performed on denoised images using the ANTs Registration SyN tool, and then the scans from week 10 were registered to the template. Using the antsApplyTransforms and fslstats tools, we obtained volume data for each structure. Then, we divide the volume of each ROI by that of the entire brain. We manually delimit each ROI using FSL’s fsleyes tool, guided by Paxinos and Watson atlas [18].

### Statistics analysis

All statistical analyses were performed using R v4.3.3. Sexual behavior parameters were not normally distributed; therefore, they were analyzed using the Friedman test, followed by Dunn’s post-hoc analysis. The data for the SIM and PPT tests were normally distributed, and the time spent in the incentive zone (SIM test) and on each compartment (PPT) were analyzed using a two-way (stimulus animal vs. sessions) repeated measures ANOVA, followed by Tukey post-hoc analysis.

Individual variations in Syp expression, brain region activation (MEMRI), and volumes concerning the treatments were analyzed per structure using the Kruskal-Wallis One-Way Analysis of Variance, followed by Dunn’s Post-Hoc tests. We used the Holm method for p-value adjustment to account for multiple comparisons. A total of 87 comparisons were controlled for.

Principal component analyses were carried out for structures of the SBN and MRS circuits using the *prcomp()* function of the *Stats* package (R Core Team 2013 stats, RRID: SCR_025968). Ninety-five percent confidence interval ellipses were drawn around the data using the *fviz_pca()* function of the *factoextra* package (Kassambara & Mundt, 2022, RRID: SCR_016692). We supplemented our principal component analysis with a linear discriminant analysis (LDA) using the lda() function in the MASS package (Venables & Ripley 2002). PCA was used to reduce the dimensionality of the dataset, while LDA was used to enhance the treatment separation.

Kendall correlation matrices and correlation tests were calculated for Syp expression, brain region activation (MEMRI), and volumes using the *cor.test* function of the *Stats* package (R Core Team 2013, RRID: SCR_025968) for the SIM and PPT tested.

For MEMRI activation, we analyzed the intensity of manganese in each group in each structure using the Software Library v6.0 (FSL). This data was not normally distributed; therefore, we analyzed it using the Kruskal-Wallis One Way Analysis of Variance, followed by Dunn Post-Hoc tests by structure in each group. Kendall correlation matrices are made using structures and correlation tests, using the cor.test function of the individual variations. Principal component analyses were carried out for structures of the SBN and MRS circuits using the prcomp() function of the Stats package (R Core Team 2013 stats, RRID: SCR_025968). Ninety-five percent confidence interval ellipses were drawn around the data using the fviz_pca() function of the factoextra package (Kassambara & Mundt, 2022, RRID: SCR_016692). We also analyze the effect of experience, evaluating the intensity of manganese in the 3 experimental sessions in each circuit in both groups. We compared both groups in each session with the Mann-Whitney U test, and week comparison for each group with the Friedman one-way repeated measurement analysis and Tukey’s post hoc.

For structural volume, the last plastic changes that we analyzed, data were obtained with Software Library v6.0 (FSL) in which the volumetric changes in each structure within each group were analyzed with the same tests mentioned above, Kruskal-Wallis One Way Analysis of Variance followed by Dunn Post-Hoc tests by group in each structure. The correlation analysis with Kendall correlation matrices and principal component analyses was carried out for structures of the SBN and MRS circuits using the prcomp() function of the Stats package (R Core Team 2013 stats, RRID: SCR_025968).

Finally, in a fourth analysis, we examined the MEMRI activation of the whole brain in weeks 1, 5, and 10 using Cohen’s effect sizes (thresholds: 0.2–1.6), comparing weeks within the same group (SIM and PPT). Then, a voxel-wise analysis was performed on denoised, spatial, and signal-intensity normalized images. Using the Glm tool from FSL, contrasts per week comparisons were created using fslmaths with a threshold of 0.95, the binarized images were corrected with Cohen’s D analysis [13]. We include the number of comparisons per test in S1 Table.

### Cohen’s size effects

To compare the whole brain activity, we determined the Cohen’s effects size (threshold 0.2–1.6) of the entire brain in weeks 1, 5, and 10 for each group as previously described [13]. Briefly, we compared the different group means by subtracting the mean voxel intensity of week 1 from that of weeks 5 and 10, and then dividing by the standard deviation of the compared weeks. Then, we used the Glm tool from FSL to perform a voxel-wise analysis on denoised, spatial, and signal-intensity normalized images. Then, the randomize function was used in the analysis. P-value-corrected images were binarized using fslmaths using the threshold of 0.95.

## Results

### Sexual behavior

No significant differences were observed in the sexual behavior parameters between the PPT and SIM groups. During the 10 weeks of testing, the percentage of subjects displaying mounts, intromissions, and ejaculations was comparable. Similarly, no consistent differences were observed in the number and latencies of mounts, intromissions, and ejaculations (S2 Table).

### SIM test

**Fig 3:**
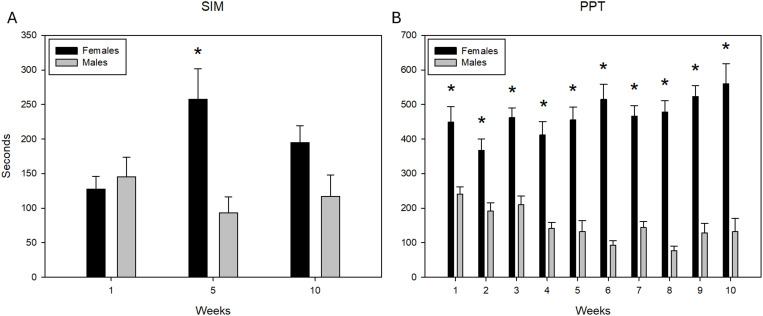
Time spent in the incentive zones of the SIM test and in the male or female compartments in the PPT. Time in seconds that the experimental subjects spent in the incentive zones of the Sexual Incentive Motivation (SIM) test in sessions 1, 5, and 10 (A) and the time the males spent in the stimulus female and stimulus male compartments in the partner preference test (PPT) during 10 sessions **(B)**. N = 10 in both tests. Data represent the mean and SEM. Two-way (stimulus animal vs. sessions) ANOVA for repeated measures, followed by Tukey test post-hoc analysis. * Significantly different from time with the male in the same session p < 0.05.

In the SIM test, we analyzed the time spent in the incentive zone of the sexually receptive female and the sexually experienced male. No significant differences were observed between the sessions [F(2, 2.985)=59, p = 0.076] but we observed significant differences between stimulus animals [F(1, 6.491)=59, p = 0.031] in session 5 (q = 5.288, p = 0.001). Moreover, in the interaction between both factors [F(2, 5.187)=59, p = 0.017], we observed that the male spent more time with the female in session 5 compared with session 1 (q = 5.661, p = 0.001) (Fig 3A). In session 10, this difference was not statistically significant (Fig 3A).

### PPT

When we analyze the time spent in the compartments with the sexually receptive female and the sexually experienced male, significant differences were observed in the session [F(9, 2.492)=179, p = 0.015], stimulus animal [F(1, 97.632)=179, p=<0.001] and in the interaction between factors [F(9, 3.964)=179, p < 0.001]. Post hoc analysis revealed that subjects spent significantly more time in the female compartment in all sessions (Fig 3B).

### Volumetric intensity of synaptophysin

**Fig 4:**
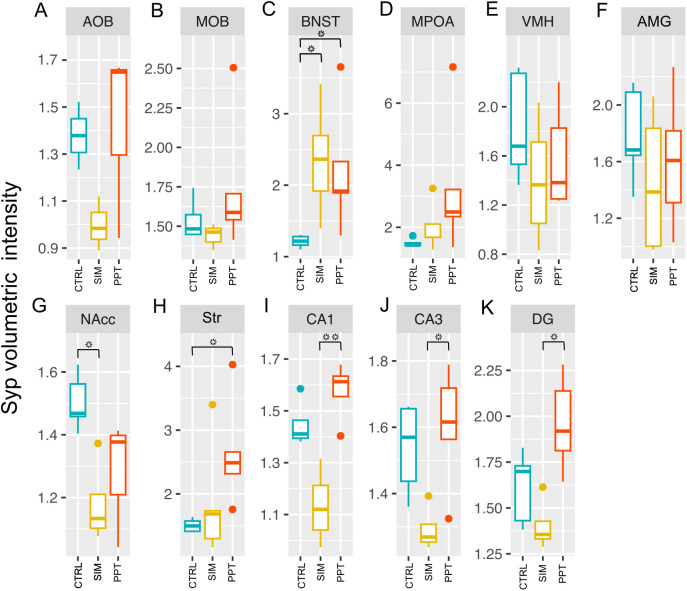
Synaptophysin volumetric intensity levels in the brain structures analyzed. Accessory Olfactory Bulb (AOB); Main Olfactory Bulb (MOB); Bed Nucleus of the Stria Terminalis (BNST); Medial Preoptic Area (MPOA); Ventromedial Hypothalamus (VMH); Amygdala (AMG); Nucleus Accumbens (NAcc); Striatum (Str), and Dentate Gyrus (DG). Kruskal-Wallis Rank Sum tests followed by Dunn Post-Hoc tests with a Holm correction of p-values (N = 15). Control group (CTRL), sexual incentive motivation (SIM), and partner preference test (PPT). *Different from the indicated group p < 0.05; **p < 0.01. Colored dots represent outlier data points.

We observed changes in Syp levels in some structures between groups (Fig 4). These changes were observed primarily in the MRS circuit, including Str, BNST, NAcc, CA1, CA3, and DG. We found increased signal intensity in the BNST (H = 8.96, df = 2, p-value = 0.01133) for the SIM (Z = −2.828, p = 0.014) and PPT (Z = −2.263, p = 0.047) groups compared to the control (Fig 4C). Also, SIM tested animals had significantly higher Syp levels in the Str compared to the control group (H = 7.02, df = 2, p = 0.0299; Z = −2.546, p = 0.033) (Fig 4H). PPT tested individuals had higher Syp levels compared to the SIM group in the CA1 (H = 8.7692, df = 2, p = 0.01247; Z = −2.942, p = 0.0097) (Fig 4I), CA3 (H = 6.6229, df = 2, p = 0.03646; Z = −2.4231, p = 0.0461) (Fig 4J) and DG (H = 8.5429, df = 2, p = 0.01396; Z = −2.922, p = 0.0104) (Fig 4K). SIM tested animals had significantly lower Syp levels in the NAcc than control individuals (H = 8.98, df = 2, p-value = 0.01122; Z = 2.869, p = 0.012) (Fig 4G).

### Synaptophysin circuit intensity analysis

**Fig 5:**
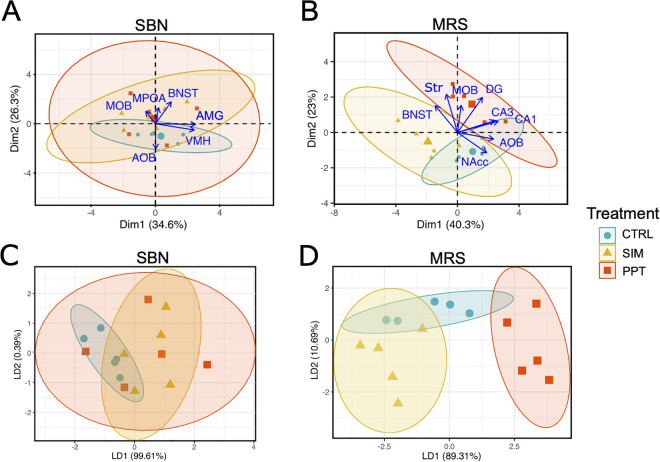
Principal Component Analysis and linear discriminant analysis of the Syp volumetric levels in the circuits. **(A)** PCA of the Socio Sexual Behavior (SBN) and the **(B)** Mesolimbic Reward System (MRS) circuits. **(C)** LDA of the Socio Sexual Behavior (SBN) N = 15. **(D)** Mesolimbic Reward System (MRS) circuits N = 15. Ellipses represent 95% confidence intervals. Large symbols in A and B represent ellipses’ centroids. Accessory Olfactory Bulb (AOB); Main Olfactory Bulb (MOB); Bed Nucleus of the Stria Terminalis (BNST); Medial Preoptic Area (MPOA); Ventromedial Hypothalamus (VMH); Amygdala (AMG); Nucleus Accumbens (NAcc); Striatum (Str) and Dentate Gyrus (DG). Control group (CTRL) blue, sexual incentive motivation (SIM) yellow, and partner preference test (PPT) red. PCA dimensions 1 and 2 (Dim 1, Dim 2), LDA discriminant axes 1 and 2 (LD1, LD2).

Principal Component Analysis and linear discriminant analysis revealed that individuals could not be segregated based on the Syp intensity levels in the SBN circuit, as principal components 1 and 2 accounted for 61% of the variance in the dataset (Fig 5A and 5C). On the other hand, SIM tested individuals were segregated from the PPT tested individuals when using the Syp levels in the MRS circuit (principal components 1 and 2 accounted for 63.3% of the variance in the data set) (Fig 5B). SIM tested individuals were completely segregated from PPT tested individuals after the LDA (Fig 5D). Control individuals remained between both treatments (Figs 5B and 5D). In order to achieve complete segregation of the SIM and PPT ellipses, AMG was excluded from the MRS dataset.

### Correlation analysis for synaptophysin expression

We found significant positive correlations for Syp expression levels between the MPOA and Str, the VMH and the AMG, the CA3 and CA1, and the DG and CA3 Table 1. The highest correlation was observed between Syp levels in AMG and VMH.

**Table 1:** Kendall’s Tau Correlation Matrix for synaptophysin expression levels across the different analyzed regions. Accessory Olfactory Bulb (AOB); Main Olfactory Bulb (MOB), Bed Nucleus of the Stria Terminalis (BNST); Medial Preoptic Area (MPOA); Ventromedial Hypothalamus (VMH); Amygdala (AMG); Nucleus Accumbens (NAcc); Striatum (Str); Dentate Gyrus (DG) and Hippocampus CA1 and CA3.

	MPOA	Str	BNST	NAcc	CA1	CA3	DG	AMG	VMH	MOB	AOB
**MPOA**											
**Str**	0.73**										
**BNST**	0.24	0.07									
**NAcc**	−0.22	−0.05	0.06								
**CA1**	0.00	0.07	−0.21	0.57							
**CA3**	0.00	0.16	−0.06	0.22	0.64*						
**DG**	0.11	0.16	−0.17	0.22	0.57	0.55*					
**AMG**	0.27	0.33	0.44	0.06	0.07	0.39	0.17				
**VMH**	0.22	0.38	0.38	0.11	0.14	0.44	0.11	0.95***			
**MOB**	0.24	0.24	−0.16	0.11	0.21	0.11	0.33	−0.06	−0.11		
**AOB**	−0.20	0.07	−0.47	0.73	0.20	0.20	0.20	−0.07	0.07	0.07	

* p < 0.05; **p < 0.01; ***p < 0.001.

### MEMRI. Structure activation analysis

**Fig 6:**
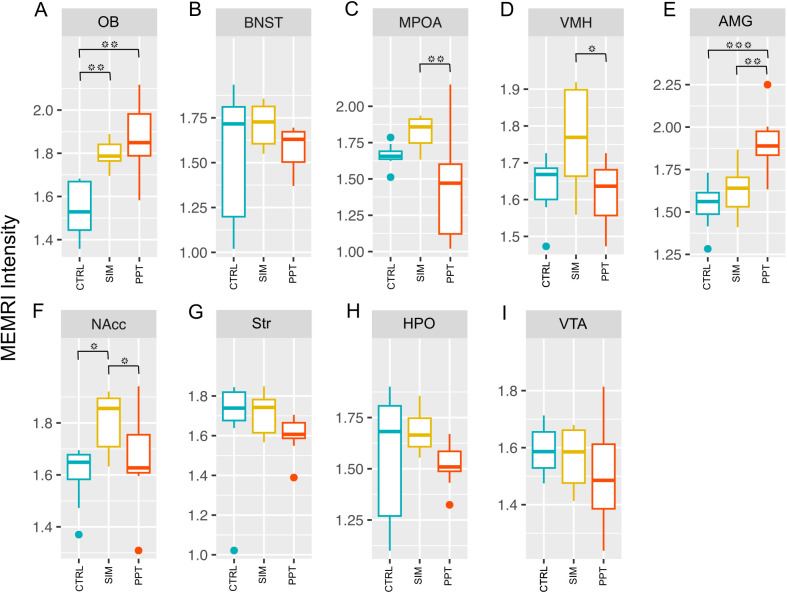
MEMRI activation levels in the brain structures analyzed at week 10. Olfactory Bulb (OB); Bed Nucleus of the Stria Terminalis (BNST); Medial Preoptic Area (MPOA); Ventromedial Hypothalamus (VMH); Amygdala (AMG); Nucleus Accumbens (NAcc); Striatum (Str); Hippocampus (HPO) and Ventral Tegmental Area (VTA). Kruskal-Wallis Rank Sum tests followed by Dunn Post-Hoc tests with a Holm correction of p-values (N = 38). Control group (CTRL), sexual incentive motivation (SIM), and partner preference test (PPT). Colored dots represent outlier data points. * p < 0.05; **p < 0.01; ***p < 0.001.

We observed changes in activation levels in week 10 between groups in some brain regions associated with the SIM and PPT tests (Fig 6). These changes were primarily observed in the SBN circuit, including OB, MPOA, AMG, and VMH. In the OB, we found an increase in signal intensity (H = 14.188, df = 2, p-value = 0.0008299) in the SIM (Z = −2.981, p = 0.0057) and PPT (Z = −3.571, p = 0.0011) groups compared to the control (Fig 6A). In the MPOA, the SIM group showed a higher activation than the PPT group (H = 11.527, df = 2, p = 0.00314; Z = −3.371, p = 0.00225) (Fig 6C). Similar results were observed in the VMH (Fig 6D), the SIM group had significantly higher activation levels compared to the PPT group (H = 6.4644, df = 2, p-value = 0.03947; Z = −2.433, p = 0.0449). In the AMG, the PPT group had significantly higher activation levels than the SIM and control groups (H = 15.423, df = 2, p-value = 0.0004476; Z = 2.992, p = 0.0055) (Fig 6E). In the NAcc, the SIM group had significantly higher activation levels (H = 8.2034, df = 2, p-value = 0.01654) compared to the control (Z = −2.601, P = 0.0279) and PPT groups (Z = −2.2833, p = 0.0448) (Fig 6F).

### MEMRI. Circuit analysis

**Fig 7:**
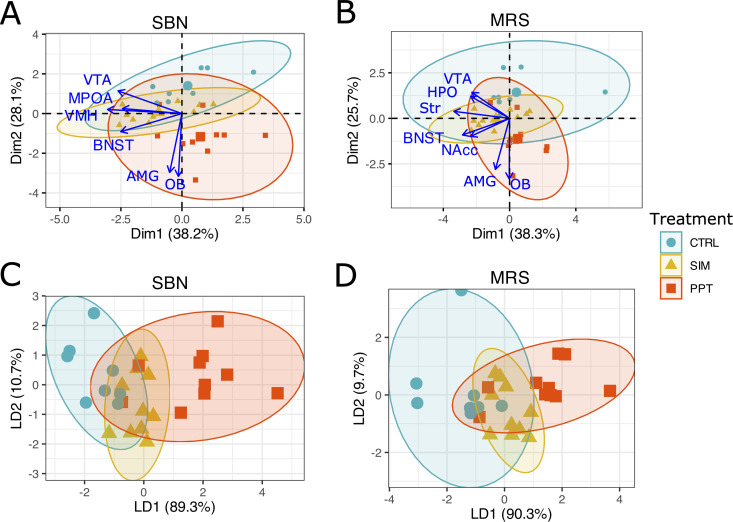
Principal Component Analysis and linear discriminant analysis of the MEMRI intensity levels. **(A)** PCA of the Socio Sexual Behavior (SBN) and the **(B)** Mesolimbic Reward System (MRS) circuits. **(C)** LDA of the Socio Sexual Behavior (SBN) and the **(D)** Mesolimbic Reward System (MRS) circuits. Ellipses represent 95% confidence intervals. Large symbols represent ellipses’ centroids. Accessory Olfactory Bulb (AOB); Main Olfactory Bulb (MOB); Bed Nucleus of the Stria Terminalis (BNST); Medial Preoptic Area (MPOA); Ventromedial Hypothalamus (VMH); Amygdala (AMG); Nucleus Accumbens (NAcc); Striatum (Str); ventral tegmental Area (VTA) and Hippocampus (HPO). Control group (CTRL), sexual incentive motivation (SIM), and partner preference test (PPT). PCA dDimensions 1 and 2 (Dim 1, Dim 2), LDA discriminant axes 1 and 2 (LD1, LD2).

PCA and LDA revealed that individuals could not be segregated based on the MEMRI intensity levels in the SBN and MRS circuits. Principal components 1 and 2 accounted for 66.3% of the variance in the SBN dataset and 64% of the variance in the MRS dataset (Fig 7).

#### Correlation analysis for MEMRI neuronal activation.

Neuronal activation was significantly correlated between different brain regions. The OB showed a positive correlation with the AMG and a negative one with the HPO. The MPOA showed significant positive correlations with 4 structures (VMH, Str, NAcc, and HPO). The BNST correlated with 5 structures (VMH, NAcc, Str, HPO, VTA). The neuronal activation of the AMG correlated positively with the OB and negatively with the HPO. The VMH showed a positive correlation with six structures (MPOA, BNST, Str, NAcc, HPO, and VTA). The Str also correlated positively with six structures (MPOA, VMH, BNST, NAcc, HPO, and VTA). The NAcc correlated positively with 4 other structures (MPOA, BNST, VMH, and Str). The neuronal activation of the HPO correlated positively with the MPOA, BNST, VMH, Str, and VTA, and it also showed a negative correlation with the OB and AMG. The VTA showed a positive correlation with the VMH, BNST, Str, and HPO. All correlations are presented in Table 2.

**Table 2:** Kendall’s Tau Correlation Matrix for activation (MEMRI). The table indicates the significance values of the correlation tests in the Olfactory Bulb (OB); Bed Nucleus of the Stria Terminalis (BNST); Medial Preoptic Area (MPOA); Ventromedial Hypothalamus (VMH); Amygdala (AMG); Nucleus Accumbens (NAcc); Striatum (Str); ventral tegmental area (VTA); and Hippocampus (HPO).

	MPOA	Str	BNST	NAcc	HPO	AMG	VMH	OB
**MPOA**								
**Str**	0.38*							
**BNST**	0.22	0.79***						
**NAcc**	0.39*	0.43**	0.39*					
**HPO**	0.32*	0.53***	0.6***	0.26				
**AMG**	0	0.02	−0.06	−0.17	−0.35*			
**VMH**	0.38*	0.67***	0.57***	0.38*	0.59***	−0.11		
**OB**	0.16	−0.05	−0.13	−0.02	−0.37*	0.41*	−0.22	
**VTA**	0.3	0.49**	0.34*	0.14	0.41*	0.16	0.50**	−0.06

*p < 0.05; **p < 0.01; ***p < 0.001

### Analysis of volume structure

**Fig 8:**
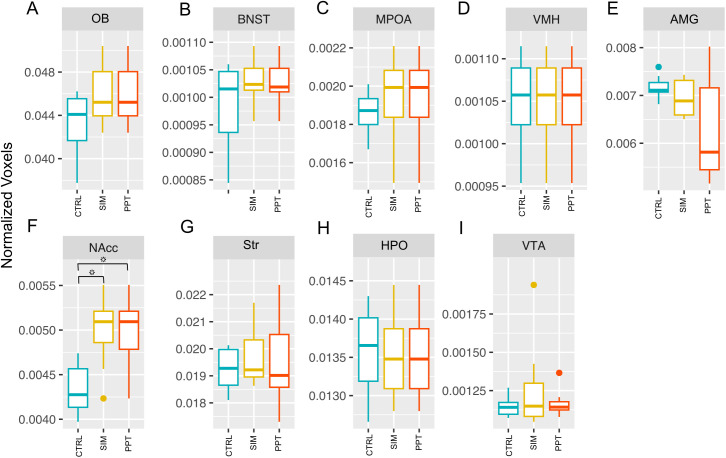
Volume measurements of normalized voxels of brain structures analyzed. Olfactory Bulb (OB); Bed Nucleus of the Stria Terminalis (BNST); Medial Preoptic Area (MPOA); Ventromedial Hypothalamus (VMH); Amygdala (AMG); Nucleus Accumbens (NAcc); Striatum (Str); Hippocampus (HPO) and Ventral Tegmental Area (VTA). Control group (CTRL), sexual incentive motivation (SIM), and partner preference test (PPT). Kruskal-Wallis Rank Sum tests followed by Dunn Post-Hoc tests with a Holm correction (N = 24). * p < 0.05.

Concerning volume analysis, the only significant difference we observed was in the NAcc (H = 8.6777, df = 2, p-value = 0.01305). The SIM (Z = −2.602, p = 0.0277) and PPT groups (Z = −2.496. p = 0.0251) had a higher volume than the control group (Fig 8F). A similar trend is observed in the OB and the MPOA, although these differences are not statistically significant (Figs 8A and 8C).

**Fig 9:**
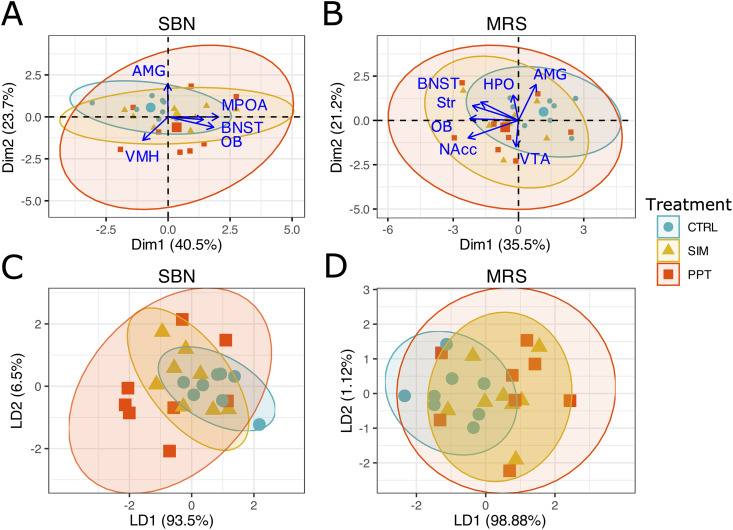
Principal Component and linear discriminant analysis of volume structure in the circuits. **(A)** PCA of the Socio Sexual Behavior (SBN) and the **(B)** Mesolimbic Reward System (MRS) circuits. **(C)** LDA of the Socio Sexual Behavior (SBN) N = 24. **(D)** Mesolimbic Reward System (MRS) circuits N = 24. Ellipses represent 95% confidence intervals. Large symbols represent ellipses’ centroids. Accessory Olfactory Bulb (AOB); Main Olfactory Bulb (MOB); Bed Nucleus of the Stria Terminalis (BNST); Medial Preoptic Area (MPOA); Ventromedial Hypothalamus (VMH); Amygdala (AMG); Nucleus Accumbens (NAcc); Striatum (Str); ventral tegmental area (VTA) and Hippocampus (HPO). Control group (CTRL) blue, sexual incentive motivation (SIM) yellow, and partner preference test (PPT) red. PCA dimensions 1 and 2 (Dim 1, Dim 2), LDA discriminant axes 1 and 2 (LD1, LD2).

PCA and LDA revealed that individuals could not be segregated based on the volumes of structures in the SBN and MRS circuits. Principal components 1 and 2 accounted for 64.2% of the variance in the SBN dataset and 56.7% of the variance in the MRS dataset (Fig 9).

In the structural volume analysis, the OB correlated with the MPOA and the Str. The MPOA correlated positively with 4 structures (OB, BNST, Str, and HPO). The BNST showed a positive correlation with the MPOA, HPO, and VTA. The VMH correlated with the VTA. The Str correlated with the OB, the MPOA, and the VTA. The HPO correlated with the MPOA, BNST, and VTA. The VTA volume correlated positively with the volume of the BNST, VMH, Str, and HPO (Table 3).

**Table 3:** Kendall’s Tau Correlation Matrix for structure volume. The table indicates the significance values of the correlation tests. In the Olfactory Bulb (OB); Bed Nucleus of the Stria Terminalis (BNST); Medial Preoptic Area (MPOA); Ventromedial Hypothalamus (VMH); Amygdala (AMG); Nucleus Accumbens (NAcc); Striatum (Str); ventral tegmental area (VTA) and Hippocampus (HPO).

	MPOA	Str	BNST	NAcc	HPO	AMG	VMH	OB
**MPOA**								
**Str**	0.43*							
**BNST**	0.39*	0						
**NAcc**	0.14	0.3	0.18					
**HPO**	0.57**	0.02	0.53**	0.14				
**AMG**	0.05	0.07	0.27	−0.23	0.15			
**VMH**	−0.28	−0.07	−0.28	0.25	−0.17	−0.21		
**OB**	0.57**	0.57**	0.25	0.28	0.14	−0.02	−0.14	
**VTA**	0.3	0.49**	0.34*	0.14	0.41*	0.16	0.50**	−0.06

* p < 0.05; **p < 0.01.

**Fig 10:**
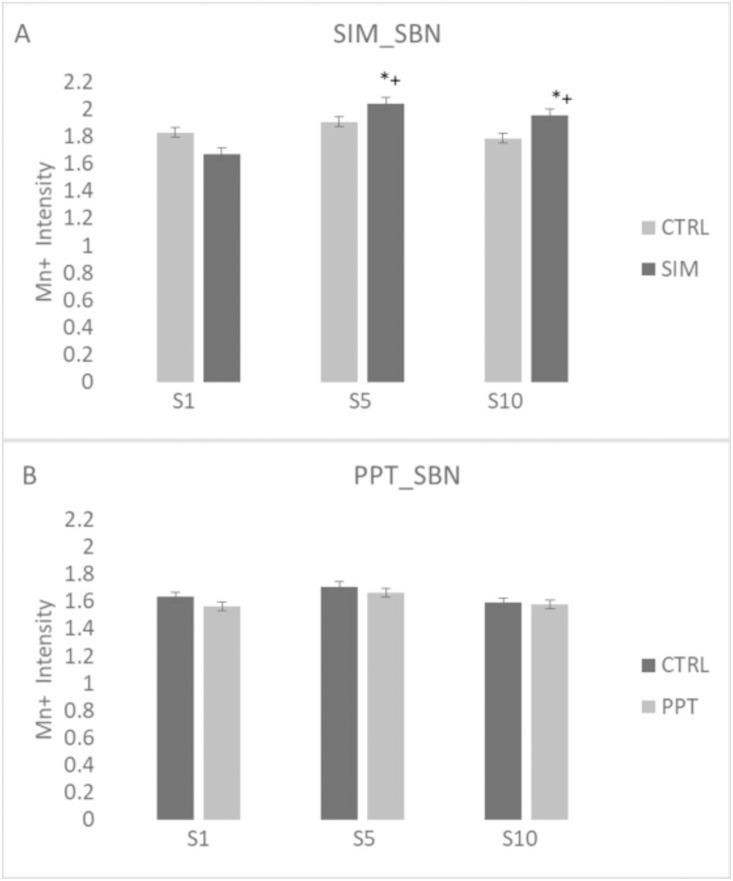
Signal intensity of Manganese (Mn +) in the Social Behavior Network (SBN) in sessions 1, 5, and 10. **(A)** Sexual Incentive Motivation (SIM) and **(B)** Partner Preference Test (PPT) groups and their respective control group (N = 10 for each group). Data represent the mean and SEM. * Significantly different from the control group; p < 0.05. + Significantly different from session 1 in the same group, p < 0.05.

We also analyzed MEMRI activation in both circuits in weeks 1, 5, and 10 to evaluate brain activation during the acquisition of sexual experience. In the SBN circuit, the SIM group showed a higher signal intensity (H = 34.614 (2), P = < 0.001) in sessions 5 (q = 7.900, p < 0.05) and 10 (q = 6.211, p < 0.05) compared to session 1. Moreover, the signal intensity was higher in sessions 5 (MWU = 698.000; T = 1518; p = 0.014) and 10 (MWU = 536.000; T = 1356; p = < 0.001) compared to the control group (Fig 10A). In the PPT group, no significant differences were observed (Fig 10B).

**Fig 11:**
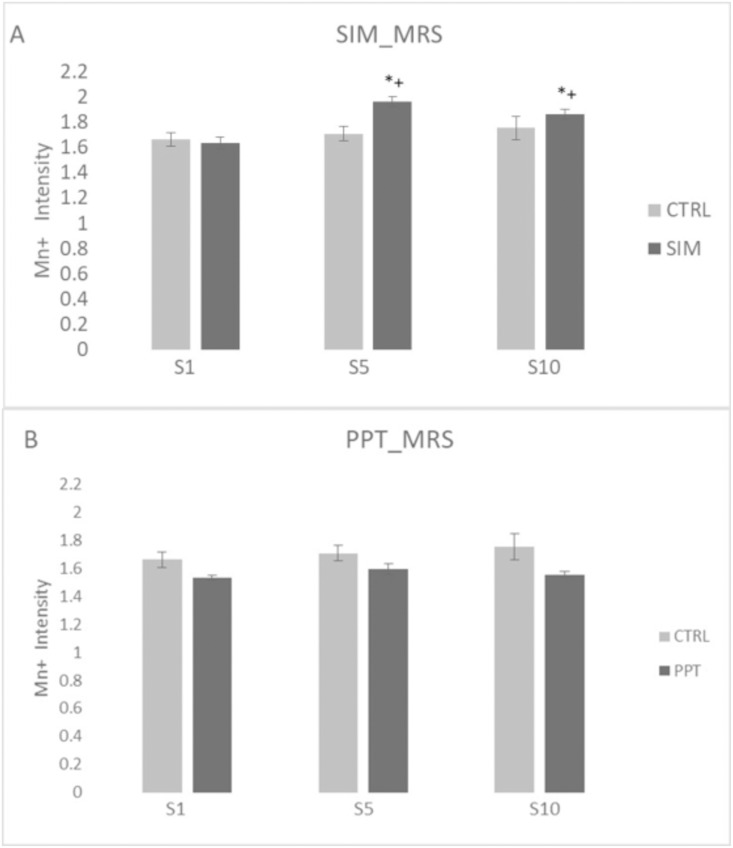
Signal intensity in the Mesolimbic Reward System (MRS) in sessions 1, 5, and 10. **(A)** Sexual Incentive Motivation (SIM) and **(B)** Partner Preference Test (PPT) groups and their respective control groups (N = 10 for experimental groups; N = 8 for control groups). Data represent the mean and SEM. * Significantly different from the control group; p < 0.05. + Significantly different from session 1 in the same group, p < 0.05.

When we analyzed the signal intensity in the MRS in weeks 1, 5, and 10, we found significant differences in the SIM group (H = 33.073 (2); P < 0.001). As in the case of the SBN circuit, the SIM group showed a higher signal intensity in weeks 5 (Q = 6.325, p < 0.05) and 10 (Q = 5.060, p < 0.05) when compared to week 1 (Fig 11A). We also found a significant increase in weeks 5 [MWU = 288.000 T = 816.000 (P = < 0.001)], and 10 [MWU = 426.000 T = 1994.000 (P = < 0.001) compared to the control group, see Fig 10A. We did not find significant differences in the PPT group (Fig 11B).

### Analysis of the whole brain activity

**Fig 12:**
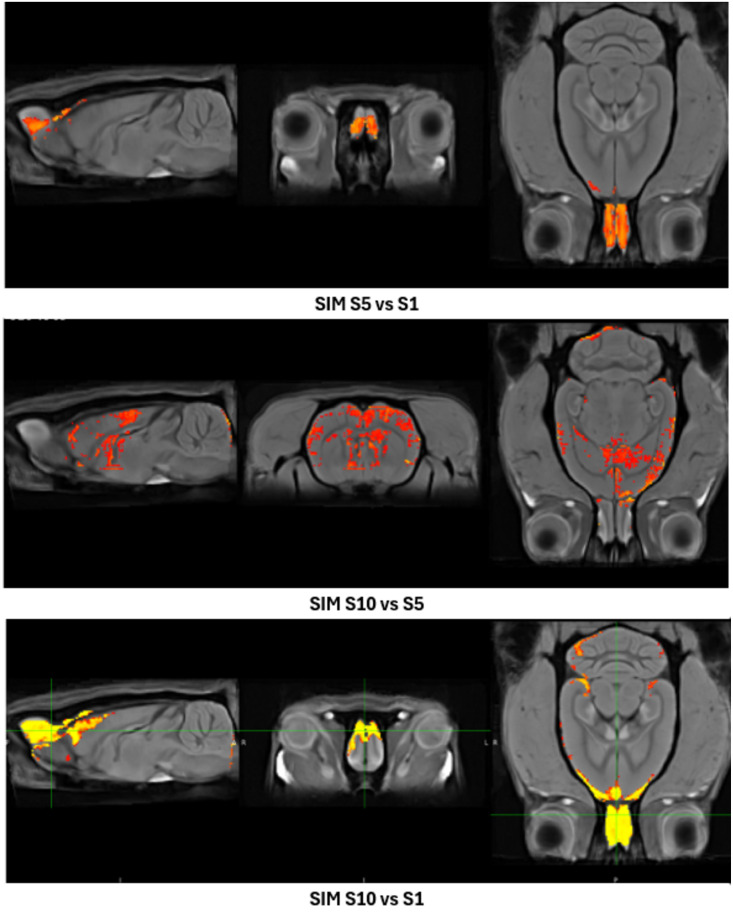
Cohen’s analysis results in the Sexual Incentive Motivation group comparing whole brain activation across the different sessions. We found a significant increase in session 5 compared to session 1 (top panel), in session 10 compared to session 5 (middle panel), and in session 10 vs session 1 (bottom panel).

We determined the Cohen’s effect size (threshold 0.2–1.6) of the whole brain activity in weeks 1, 5, and 10 to analyze brain activity between weeks. For that purpose, the mean of week 1 was subtracted from the mean of week 10 and then divided by the standard deviation of week 1. The same procedure was applied for week 5. This was also carried out to compare weeks 5 and 10. In the SIM group (Fig 12), we observed a significant increase in session 5 compared to session 1 (top panel), in session 10 compared to session 5 (middle panel), and in session 10 vs session 1. We did not find significant differences in the PPT group.

**Fig 13:**
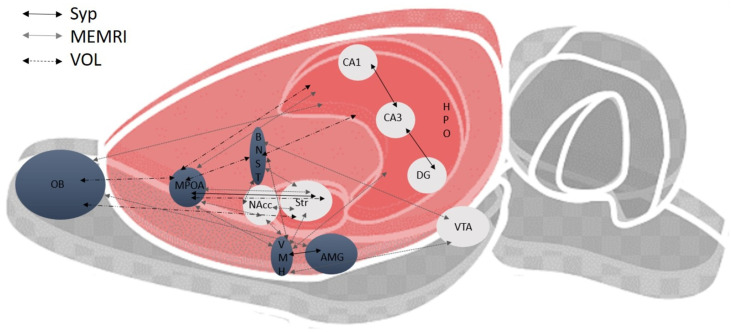
The sagittal section of the brain depicts the correlations found in the three plastic changes analyzed by MEMRI. Plastic changes were: intensity of synaptophysin (Syp) expression, increased signal intensity by MEMRI, and volume analysis. Gray areas (SBN); white areas (MRS). Olfactory bulb (OB); medial preoptic area (MPOA); bed nucleus of the stria terminalis (BNST); nucleus accumbens (NAcc); ventromedial hypothalamus (VMH); striatum (Str); amygdala (AMG); hippocampus (CA1, CA3, DG); ventral tegmental area (VTA); manganese-enhanced magnetic resonance imaging (MEMRI); volume (VOL).

A summary of the positive correlations described above for Syp expression, MEMRI activation, and volume changes indicates that the MPOA and the Str had 9 and 10 correlations with different structures, followed by the BNST, VMH, HPO, and VTA, which correlated with 8 different structures (Fig 13).

## Discussion

Our results indicate that males in the SIM and PPT clearly preferred the females during the different weeks of testing, reflecting the acquisition of sexual experience across sessions. The time spent in the female compartment increased because the female is an incentive for the male rat [2]. Partner preference induced a higher expression of Syp in the BNST and Str vs the control group. In all regions analyzed of the HPO (CA1, CA3, and DG), the PPT group showed a higher intensity than the SIM group, indicating that the execution of sexual behavior produces long term synaptic modifications in brain regions of the MRS. The BNST also showed an increase in Syp expression in both the SIM and PPT, further confirming the importance of this brain region as an interface between the SBN and the MRS.

With MEMRI, we also observed a higher signal intensity in the partner preference test in the OB and AMG. Sexual incentive motivation induced higher activation in the OB compared to CTRL and in the NAcc compared to CTRL and PPT. Moreover, SIM induced a higher level of activation than PPT in the MPOA and VMH. The changes observed in the present study were analyzed at the end of the 10 weeks of testing. Further studies will need to address whether the changes start occurring in previous weeks as the subjects acquire sexual experience.

Syp expression and other synaptic vesicle proteins are implicated in mechanisms of cellular plasticity underlying learning [19–21]. The expression of Syp showed that the acquisition of sexual experience caused significant changes in the Str (PPT vs CTRL) in the HPO (PPT vs SIM) and the NAcc (CTRL vs SIM), all regions belonging to the MRS circuit. It is suggested that the observed differences in Syp volumetric intensity and the MEMRI techniques used between the groups are a direct reflection of the different behavioral testing conditions, *per se*. Although both the SIM and PPT groups received copulation sessions, the SIM group had intermediate sessions in which interaction with the stimulus was somewhat limited through a wire mesh enclosure, allowing the subject to see, smell, and hear the stimulus animals. It could be argued that the difference in the number of copulation sessions could affect the results; however, our data demonstrate that three additional weeks of sexual experience in the PPT group compared to the SIM group does not make a difference in cumulative experience (S2 Table). The observed differences in Syp volumetric intensity, in the SIM and the PPT groups, reflect the importance of acquiring sexual experience to observe long lasting brain modifications. Two neurotransmitters that could modulate these changes are dopamine (DA) and opioids. Sexual behavior activates and/or sensitizes the nigrostriatal and mesolimbic pathways, which are known to participate in the regulation of motivated behaviors such as sex in the processing of rewards and sensorimotor integration [22]. Evidence indicates that DA participates in wanting but not liking different stimuli, where wanting is mediated by a general cerebral system related to DA, and liking, a hedonic process, is mediated by opioids [23]. The dopaminergic circuits of the striatum do not participate in the rewarding aspects of sexual behavior but produce a general system activation [24]. Opioids mediate the rewarding properties of mating in males and females [25–27]. Future research will investigate the modifications observed in the present study and determine whether they are associated with changes in the neurotransmitters mentioned above.

Due to its hedonic nature, the rewarding aspects of sexual experience ensure that the behavior will be repeated. Participation in the processing of hedonic or motivational value is induced by the NAcc shell [28–30], which receives information from limbic structures. On the other hand, the learning process and actions directed toward a particular objective are facilitated by the central part of the NAcc, as it projects to motor areas to guide behavior [31,32]. The two subregions of the NAcc are linked to value-based associative processing [32,33]. The motivational and motor parts are necessary for flexible behavior to respond appropriately to a particular situation. Behavioral flexibility is essential because animals, such as rodents, must know how to adapt to complex and changing environments, including natural ones. By flexible behavior, we refer to all responses that can be modified according to their context or environmental demands [34–36].

McGinty and collaborators [35] refer to flexible and inflexible approach tasks as those in which a novel locomotor sequence is either required or not required to achieve a goal. An inflexible task can be seen as a habitual behavior where performing specific actions leads to a reward. It has been proposed that habitual behavior does not depend on the NAcc DA [34,36]. This could explain the unexpected decrease in Syp in the SIM group compared to the control group in the NAcc. Further studies should be carried out, separating the core and shell as they have different functions. The NAcc shell is linked to reward processing, as well as emotional and motivational processes, while the core is related to motor functions [37]. Another possible explanation is the time window we used to evaluate Syp expression. We sacrificed the animals after 10 weeks of repeated testing. Initial changes could occur in different brain regions at the beginning of testing when animals do not have much sexual experience. Only future studies could address this issue.

The increase in Syp intensity in the CA1, CA3, and DG in the PPT group compared to the SIM group could be associated with the involvement of the HPO in different types of memories, suggesting that socio-sexual memory is, at least in part, stored in the HPO [38,39]. On the other hand, the release of endogenous neurotrophic factors induces an increase in Syp in brain regions, including the HPO, DG, and neocortex [40,41]. Some studies have shown that a higher amount of Syp in regions such as the HPO and the DG is positively related to better performance in cognitive activities, such as learning and memory, even in senile stages [42], decision-making functions, and in lower animals to respond to sexual invitations by detecting pheromones, and olfactory discrimination, among others [43]. Individuals tested for SIM were completely segregated in the principal components analysis from those tested for PPT based on Syp levels of the MRS circuit (Fig 5). Regions of the HPO drove this segregation: CA1, CA3, and DG behaved very similarly, with higher Syp levels in PPT tested individuals and lower levels for SIM tested individuals. In NAcc, interestingly, we observed an opposite effect to that which occurred in the expression of Syp. We observed a significant increase in activation in the SIM group compared to the PPT and the control group. Possibly because the connections are refined, and there is no need for more expression of synaptic proteins. However, the activation of the remaining synapses is still active. The VMH and AMG are rich in steroid receptors, so they are activated by the partner’s odor and processed as a reinforcing value during sexual behavior.

The analysis of signal intensity by MEMRI revealed higher activation in structures belonging to the SBN circuit, OB, and AMG in the SIM and PPT groups compared to the control group. In the MPOA and VMH, SIM induced a higher activation than the PPT group. The results are consistent with previous studies in which we demonstrated a significant increase in the OB in the SIM group [13]. We also found significant differences in the NAcc (MRS); the SIM group showed a higher activation than the control and PPT groups. After analyzing the circuits over the weeks, we observed a significant increase in signal intensity in the SIM group in both circuits. No changes were observed in the PPT group, indicating that even though the animals already have sexual experience, sexual motivation is necessary for an optimal display of the behavior, even after 10 weeks of testing. It is not surprising that some of the structures related to sexual motivation, such as the MPOA and BNST [44,45], showed the highest number of correlations (Fig 13) with other brain structures. However, we cannot rule out the possibility that the changes observed between PPT and SIM are due to the difference in the number of weeks of experience, 7 in SIM and 10 in PPT.

Regarding the volume analysis, we observed a significant increase in the NAcc only in the SIM and PPT groups compared to the control group. These changes could reflect the accumulated experience associated with the plastic changes in previous weeks: 10 weeks in the PPT group and 7 in the SIM group. Both groups were different from the control. These results are consistent with Cohen’s d analysis, which showed changes in sessions 5 and 10 compared to session 1.

Several studies have shown that sexual experience can induce significant changes in neuroanatomy. Our finding indicates an increase in NAcc volume by MEMRI. In contrast, recent research in mice on voluntary exercise wheels did not find an increase in NAcc volume but did find an increase in other evaluated structures, such as the red nucleus and the HPO [46]. This result suggests that, unlike physical exercise, which did not generate changes in this brain region, sexual experience could be associated with greater neuroanatomical plasticity, possibly because it requires greater recruitment of sensory systems, since the animal needs to discriminate between the two stimuli to which it has access and, at the same time, perform the motor part.

The NAcc is fundamental in driving goal-directed actions, integrating neuromodulatory input to optimize motivated behavioral outcomes. Long-term changes in synaptic strength within the NAcc underlie experience-dependent neural plasticity. These changes include intricate molecular epigenetic, biochemical, electrophysiological, and morphological changes in individual neurons, ultimately reshaping synaptic function [47,48]. The volumetric change we observed in NAcc could likely be associated with an increase in various plastic changes, such as those mentioned above, which leads to an increase in the activity shown in the MEMRI analysis. Future studies are required to elucidate this relationship. Several studies have shown that sexual experience can induce significant changes in neuroanatomy. Our finding indicates an increase in NAcc volume by MEMRI. In contrast, recent research in mice on voluntary exercise wheels did not find an increase in NAcc volume but did find an increase in other evaluated structures, such as the red nucleus and the HPO [46]. These results suggest that, unlike physical exercise, which did not generate changes in this brain region, sexual experience could be associated with greater neuroanatomical plasticity, possibly because it requires greater recruitment of sensory systems, since the animal needs to discriminate between the two stimuli to which it has access and, at the same time, perform the motor part.

It is important to remember that techniques such as functional magnetic resonance imaging (fMR) are based on detecting variations in oxygenated blood levels due to brain activity through the BOLD signal. It is clear that sexual activity causes an increase in blood flow to brain areas, as also occurs with other activities such as exercise. This could reflect increased activity and contribute to an increase in the volume of this structure. Increased flow is essential to meet the metabolic needs of neurons activated by sexual activity. However, in the technique we used (MEMRI), manganese is used as a calcium analogue. When neuronal activity increases, manganese accumulates in active neurons. At least until a few years ago, MEMRI was the only fMR method capable of mapping brain activation in vivo, independent of the indirect hemodynamic changes used in fMR [49].

The different correlation matrices indicate that in Syp expression, we found 4 correlations, 20 correlations when analyzing increased signal activity by MEMRI, and 6 with volume analysis. It is clear that the structures of the two circuits undergo the three plastic changes. However, we can observe that the MPOA is one of the structures that shows the highest number of correlations (Fig 12), further confirming the vital role of this structure in controlling male sexual behavior.

## Conclusion

Our results are consistent with our hypothesis, as we found a higher number of plastic changes in the SIM group compared to PPT in the MRS circuit, as well as a higher number of plastic changes in structures belonging to SBN in both groups. The acquisition of sexual experience induces different plastic changes in the brain structures of the SBN and MRS circuits. Syp expression increased in most structures of the MRS and the BNST. An increase in signal intensity was observed in most areas of the SBN circuit and the NAcc. In the volume results, we observed a significant increase in the NAcc in both groups vs the control group. On the other hand, our correlation results confirm that the two circuits participate in the acquisition of sexual experience. The structures that showed the highest number of correlations were the MPOA and Str. The results of the present experiment confirm that numerous neuroplastic changes (Syp expression, increased signal intensity measured by MEMRI, and volume increase) occur in both MRS and SBN circuits during the acquisition of sexual experience in motivation and execution. The expression of a motivated (sexual) behavior induces micro and macro neuroplastic changes in brain regions and circuits necessary for the expression of sexual behavior.

## Supporting information

S1 Table Number of comparisons performed in the statistical test. (DOCX)

S2 Table Behavioral parameters in SIM and PPT groups during the 10 weeks of testing. NA: does not apply. *Different from the PPT group.
(DOCX)
