# Supplementary material for: Micro and macro structural brain plastic changes induced by sexual experience in male rats
Source: PLoS One. 2025 Oct 28;20(10):e0334959. doi: 10.1371/journal.pone.0334959 (PMC12561951; doi:10.1371/journal.pone.0334959)
Supplement: S1 Table — (DOCX) [file pone.0334959.s001.docx]

Table S2. Number of comparisons performed in the statistical test.

| **Variable** | **Number of structures analyzed** | **Experimental groups** | **Statistical tests** | **Post-hoc tests** | **p value correction** |
| --- | --- | --- | --- | --- | --- |
| Synaptophysin | 11 | 3 | Kruskal-Wallis One Way Analysis of Variance | Dunn | Holm |
| MEMRI | 9 | 3 | Kruskal-Wallis One Way Analysis of Variance | Dunn | Holm |
| Volume | 9 | 3 | Kruskal-Wallis One Way Analysis of Variance | Dunn | Holm |
