# Supplementary material for: Micro and macro structural brain plastic changes induced by sexual experience in male rats
Source: PLoS One. 2025 Oct 28;20(10):e0334959. doi: 10.1371/journal.pone.0334959 (PMC12561951; doi:10.1371/journal.pone.0334959)
Supplement: S2 Table — NA: does not apply. *Different from the PPT group. (DOCX) [file pone.0334959.s002.docx]

S1 Table. Behavioral parameters in the Sexual Incentive Motivation (SIM) and Partner Preference Test (PPT) groups during the 10 weeks of testing. NA: does not apply.

*Different from the PPT group.

|  | Percentages | | | | | |
| --- | --- | --- | --- | --- | --- | --- |
|  | Mount | | Intromission | | Ejaculation | |
| Week | SIM | PPT | SIM | PPT | SIM | PPT |
| 1 | NA | 80 | NA | 70 | NA | 20 |
| 2 | 100 | 70 | 90 | 40 | 20 | 30 |
| 3 | 100 | 80 | 100 | 80 | 50 | 40 |
| 4 | 100 | 100 | 100 | 90 | 60 | 40 |
| 5 | NA | 90 | NA | 80 | NA | 80 |
| 6 | 100 | 90 | 90 | 90 | 50 | 80 |
| 7 | 100 | 100 | 90 | 100 | 80 | 60 |
| 8 | 100 | 100 | 100 | 100 | 60 | 70 |
| 9 | 100 | 80 | 90 | 80 | 80 | 50 |
| 10 | NA | 70 | NA | 70 | NA | 60 |
|  | Number | | | | | |
|  | Mount | | Intromission | | Ejaculation | |
| Week | SIM | PPT | SIM | PPT | SIM | PPT |
| 1 | NA | 2.6 ± 1.2 | NA | 5.5 ± 2.32 | NA | 0.9 ± 0.31 |
| 2 | 9.9 ± 1.3 * | 2.1 ± 0.9 | 6.7 ± 3.02 | 3.7 ± 1.66 | 0.3 ± 0.213 | 0.6 ± 0.16 |
| 3 | 8.9 ± 1.9 | 3.5 ± 1.3 | 12.50 ± 1.98 | 7.8 ± 1.81 | 0.7 ± 0.3 | 1.1 ± 0.31 |
| 4 | 9.3 ± 2.5 | 5.2 ± 1.1 | 16 ± 1.92 * | 10.5 ± 2.12 | 1 ± 0.39 | 0.73 ± 0.26 |
| 5 | NA | 6.4 ± 2.02 | NA | 13.4 ± 2.45 | NA | 1.2 ± 0.24 |
| 6 | 7.3 ± 2.14 * | 8.1 ± 2.02 | 14.33 ± 2.78 | 11.3 ± 1.46 | 1 ± 0.36 | 1.2 ± 0.29 |
| 7 | 5.8 ± 1.91 * | 4.6 ± 1.3 | 18.99 ± 2.22 | 20.1 ± 2.81 | 0.9 ± 0.37 | 1 ± 0.19 |
| 8 | 13.7 ± 8.96 * | 4.1 ± 1.41 | 16.4 ± 2.68 | 12.3 ± 1.94 | 0.9 ± 0.34 | 1.13 ± 0.23 |
| 9 | 7.10 ± 2.08 * | 4.4 ± 1.49 | 13.7 ± 2.69 | 9.1 ± 1.96 | 1 ± 0.36 | 1.4 ± 0.26 |
| 10 | NA | 3.6 ± 1.25 | NA | 7.3 ± 2.05 | NA | 1.4 ± 0.25 |
|  | Latency | | | | | |
|  | Mount | | Intromission | | Ejaculation | |
| Week | SIM | PPT | SIM | PPT | SIM | PPT |
| 1 | NA | 433.9± 3.63 | NA | 496.1±100.6 | NA | 774.7± 83.74 |
| 2 | 190.6 ± 40.25 | 501.8 ± 10.8 | 872.3 ±169.93 | 647.9±111.7 | 1652.9±143.7* | 781 ± 73.24 |
| 3 | 291.6±151.51 | 351.5± 3.69 | 480.4 ± 160.17 | 412.5±113.2 | 1332.9±165.3* | 683.± 110.71 |
| 4 | 143.2 ±41.20 | 155.8 ± 5.78 | 318.4 ± 103.12 | 193.7±88.57 | 1320.8±171.23 | 706.9 ± 93.63 |
| 5 | NA | 209.3±93.2 | NA | 254.5±110.0 | NA | 514.4 ± 8.98 |
| 6 | 74.3 ± 17.59 | 189.8 ± 7.21 | 297.1 ± 170.76 * | 203.4±85.27 | 1255.7±206.1* | 578.7 ± 90.27 |
| 7 | 179.2 ± 8.53 | 118.2 ± 6.86 | 362.2 ± 77.12 | 180.8±66.76 | 1228.4±184.65 | 628.2 ± 91.74 |
| 8 | 247.1 ±96.15 | 64.3 ± 15.76 | 493.4 ± 186.80 * | 129.1±53.74 | 1182.9±196. * | 516.8 ± 95.67 |
| 9 | 141.1 ± 41.70 | 237.1±112.6 | 417.5 ± 167.95 * | 330.5±123.6 | 1082.2±142.8* | 634.9 ± 96.21 |
| 10 | NA | 354.6±124.3 | NA | 363.7±122.8 | NA | 537.4 ± 06.66 |
